# Impregnated and Co-precipitated Pd–Ga_2_O_3_, Pd–In_2_O_3_ and Pd–Ga_2_O_3_–In_2_O_3_ Catalysts: Influence of the Microstructure on the CO_2_ Selectivity in Methanol Steam Reforming

**DOI:** 10.1007/s10562-018-2491-4

**Published:** 2018-08-03

**Authors:** Christoph Rameshan, Harald Lorenz, Marc Armbrüster, Igor Kasatkin, Bernhard Klötzer, Thomas Götsch, Kevin Ploner, Simon Penner

**Affiliations:** 1 Institute of Physical Chemistry, University of Innsbruck, Innrain 52c, 6020 Innsbruck, Austria; 2 Institute of Chemistry, Technical University Chemnitz, Straße der Nationen 62, 09111 Chemnitz, Germany; 3 Department of Inorganic Chemistry, Fritz-Haber Institute of the Max-Planck-Society, Faradayweg 4-6, 14195 Berlin, Germany; 4 Present Address: Saint Petersburg State University, Universitetskaya nab. 7-9, St. Petersburg, Russia 199034; 5 Present Address: Institut für Materialchemie, Technische Universität Wien, Getreidemarkt 9/BC/01, 1060 Vienna, Austria

**Keywords:** X-ray diffraction, High-resolution electron microscopy, Catalysis, Intermetallic compound, Hydrogen reduction, Catalyst activation

## Abstract

**Abstract:**

To focus on the influence of the intermetallic compound—oxide interface of Pd-based intermetallic phases in methanol steam reforming (MSR), a co-precipitation pathway has been followed to prepare and subsequently structurally and catalytically characterize a set of nanoparticulate Ga_2_O_3_- and In_2_O_3_-supported GaPd_2_ and InPd catalysts, respectively. To study the possible promoting effect of In_2_O_3_, an In_2_O_3_-doped Ga_2_O_3_-supported GaPd_2_ catalyst has also been examined. While, upon reduction, the same intermetallic compounds are formed, the structure of especially the Ga_2_O_3_ support is strikingly different: rhombohedral and spinel-like Ga_2_O_3_ phases, as well as hexagonal GaInO_3_ and rhombohedral In_2_O_3_ phases are observed locally on the materials prior to methanol steam reforming by high-resolution transmission electron microscopy. Overall, the structure, phase composition and morphology of the co-precipitated catalysts are much more complex as compared to the respective impregnated counterparts. However, this induces a beneficial effect in activity and CO_2_ selectivity in MSR. Both Ga_2_O_3_ and In_2_O_3_ catalysts show a much higher activity, and in the case of GaPd_2_–Ga_2_O_3_, a much higher CO_2_ selectivity. The promoting effect of In_2_O_3_ is also directly detectable, as the CO_2_ selectivity of the co-precipitated supported Ga_2_O_3_–In_2_O_3_ catalyst is much higher and comparable to the purely In_2_O_3_-supported material, despite the more complex structure and morphology. In all studied cases, no deactivation effects have been observed even after prolonged time-on-stream for 12 h, confirming the stability of the systems.

**Graphical Abstract:**

The presence of a variety of distinct supported intermetallic InPd and GaPd_2_ particle phases is not detrimental to activity/selectivity in methanol steam reforming as long as the appropriate intermetallic phases are present and they exhibit optimized intermetallic-support phase boundary dimensions.

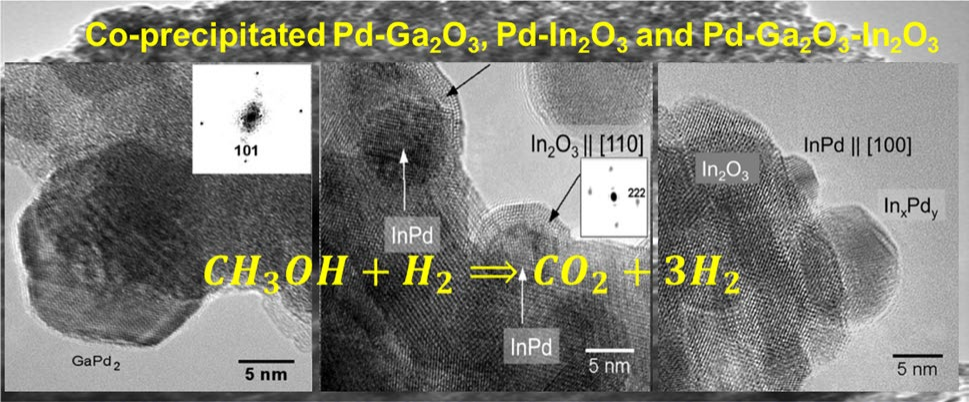

## Introduction

Pd-based intermetallic compounds have long been in the focus of research due to their outstanding catalytic properties in methanol steam reforming [1–5]. The associated high CO_2_-selectivity has tentatively been ascribed to the general presence of the intermetallic compound after reduction in hydrogen, whose electronic structure mimics that of the technologically used Cu/ZnO catalysts [6]. It is now widely accepted that the mere presence of the intermetallic compound alone is not sufficient to explain the high CO_2_ selectivity, but rather, the close contact to the oxide phase is a prerequisite for efficient water activation, the crucial step in obtaining high CO_2_ selectivities [7–9]. Among the studied systems, GaPd_2_/Ga_2_O_3_ [10–16], ZnPd/ZnO [5, 17–21] and InPd/In_2_O_3_ [22–24] have been scrutinized most and many of their structural, physico-chemical and catalytic properties have been already determined satisfactorily. As the simultaneous presence of both intermetallic compound and (partially reduced) oxide supports (monoclinic Ga_2_O_3_, hexagonal ZnO and cubic In_2_O_3_, respectively) is of utmost importance to induce a bifunctional synergism and, thus, to obtain high CO_2_ selectivities, to search for synthesis methods in order to obtain a potentially larger intermetallic-oxide interface concentration is imperative. So far, apart from thin film or other model catalyst approaches [4, 7, 8, 14, 19–22], preparation of those catalysts is basically performed using standard incipient wetness impregnation pathways.

In this work, to increase the supposedly catalytically active interface, we follow a Pd and Ga_2_O_3_ and In_2_O_3_ co-precipitation approach using nitrate precursor solutions, respectively. In due course, the structure and catalytic properties are directly compared to their impregnated counterparts. The present work also focuses on the possible difference between using either Ga_2_O_3_ or In_2_O_3_ as active catalyst support, because Ga_2_O_3_-containing catalysts are known to exhibit a significantly lower CO_2_ selectivity as compared to their In_2_O_3_-containing counterparts. This task is tackled by deliberately promoting a Pd–Ga_2_O_3_ catalyst with In_2_O_3_ in the co-precipitation process. Additionally, we present long-term activity measurements for the entire set of catalysts to elucidate deactivation, an undesired catalytic property that has not been addressed in detail for this class of materials so far. Special attention will be given to a detailed comparison of the structure and morphology of both (inter)metallic and oxide particles before and after the methanol steam reforming treatment, thus extensive high-resolution electron microscopy experiments are an integral part of the work.

## Experimental

### Catalyst Preparation

In order to suppress a potential influence of the preparation routine on the catalytic properties, the synthesis protocols were kept as similar as possible for all catalysts. This particularly refers to the way Pd is introduced, as well as to the solvents and precipitation agents used.

For the co-precipitated Pd/Ga_2_O_3_ catalyst, 100 mg Pd (Goodfellow Pd foil 99.99%) were dissolved in a mixture of 5 mL HNO_3_ (65%) and 1 mL HCl (37%) while gently heating. Subsequently, the volume was increased with distilled H_2_O to 50 mL. Separately, 1.5 g Ga (Goodfellow Ga metal 99.9999%) were dissolved in HCl (37%) at 373 K. Both solutions were unified and diluted to 100 mL total volume using distilled H_2_O. Afterwards, NaOH (5%) was added dropwise at 353 K until a pH value of 7–8 was reached. The resulting precipitate was allowed to age overnight and subsequently filtrated and dried at 373 K. Remaining Cl was removed by thorough washing. To obtain the pre-catalyst, the powder was calcined in air at 773 K for 4 h (which also removes the remnants of HNO_3_ decomposition).

Similarly, the respective co-precipitated Pd/In_2_O_3_ and Pd–Ga_2_O_3_–In_2_O_3_ catalysts were synthesized. For the former, In metal foil (Goodfellow 99.999%) was dissolved in HNO_3_ and In(OH)_3_ precipitated by addition of NaOH (5%). 2.4 g In(OH)_3_ were then dissolved in 5 mL HCl (37%) and diluted using distilled H_2_O up to 20 mL total volume. The Pd-containing solution (preparation exactly as above) was then added and the unified solution treated as the Pd–Ga_2_O_3_ catalyst above.

The catalyst containing both Ga_2_O_3_ and In_2_O_3_ was prepared by unifying the Pd- and Ga_2_O_3_-containing solutions (preparation as above) with the respective In(OH)_3_ solution (0.24 g in 5 mL HCl 37%). Further aging, filtration and calcination were performed as above. Impregnated Pd/Ga_2_O_3_ and Pd/In_2_O_3_ have been prepared following a classical wet impregnation technique detailed elsewhere [10, 12].

**Fig. 1:**
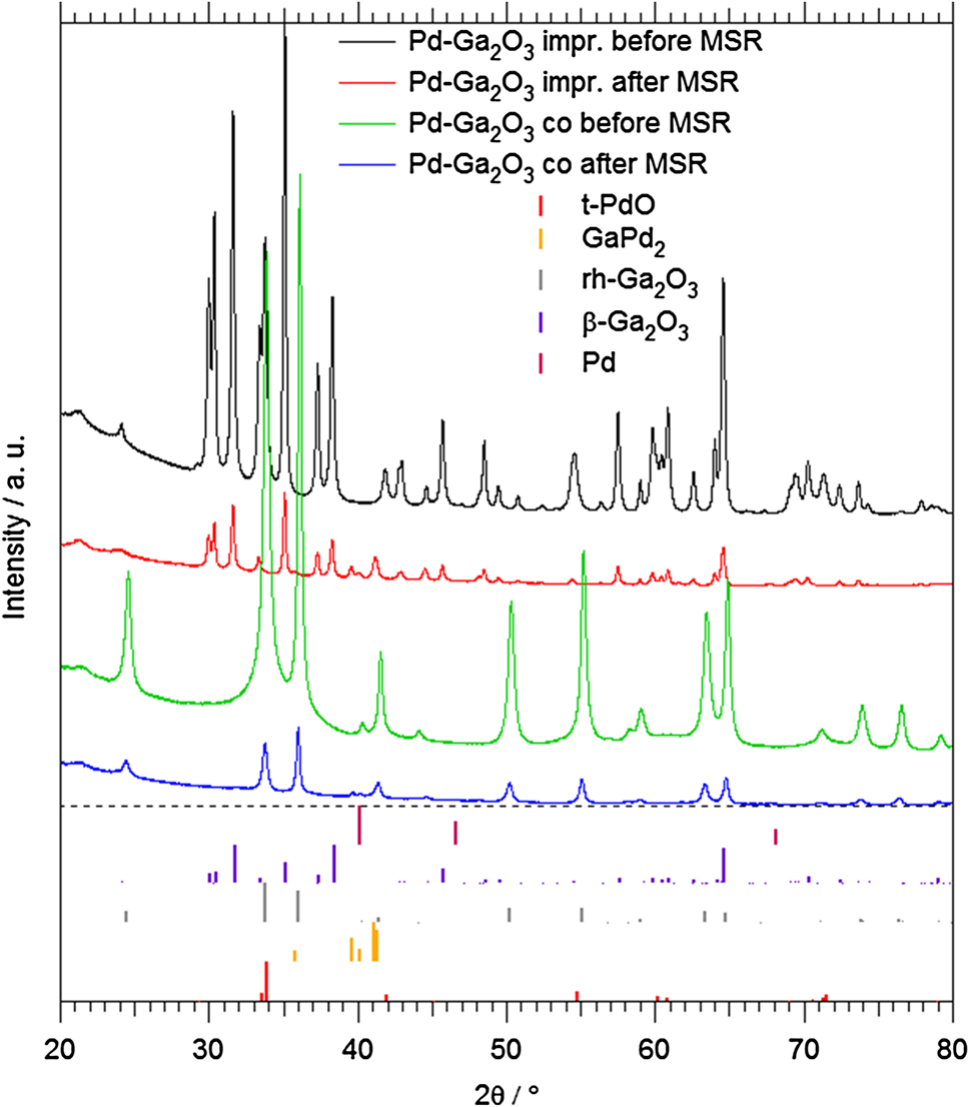
PXRD data of impregnated and co-precipitated Pd-Ga_2_O_3_ before oxidation and after the catalytic testing. Reference diffractograms for tetragonal PdO (#43-1024), orthorhombic GaPd_2_ (#50-1443), rhombohedral Ga_2_O_3_ (#43-1013), monoclinic Ga_2_O_3_ (#43-1012) and cubic Pd metal (#46-1043) for phase analysis are shown as vertical bars. Data of the H_2_ pre-reduced state (after calcination and before MSR) are almost identical to those after MSR and are therefore not shown

**Fig. 2:**
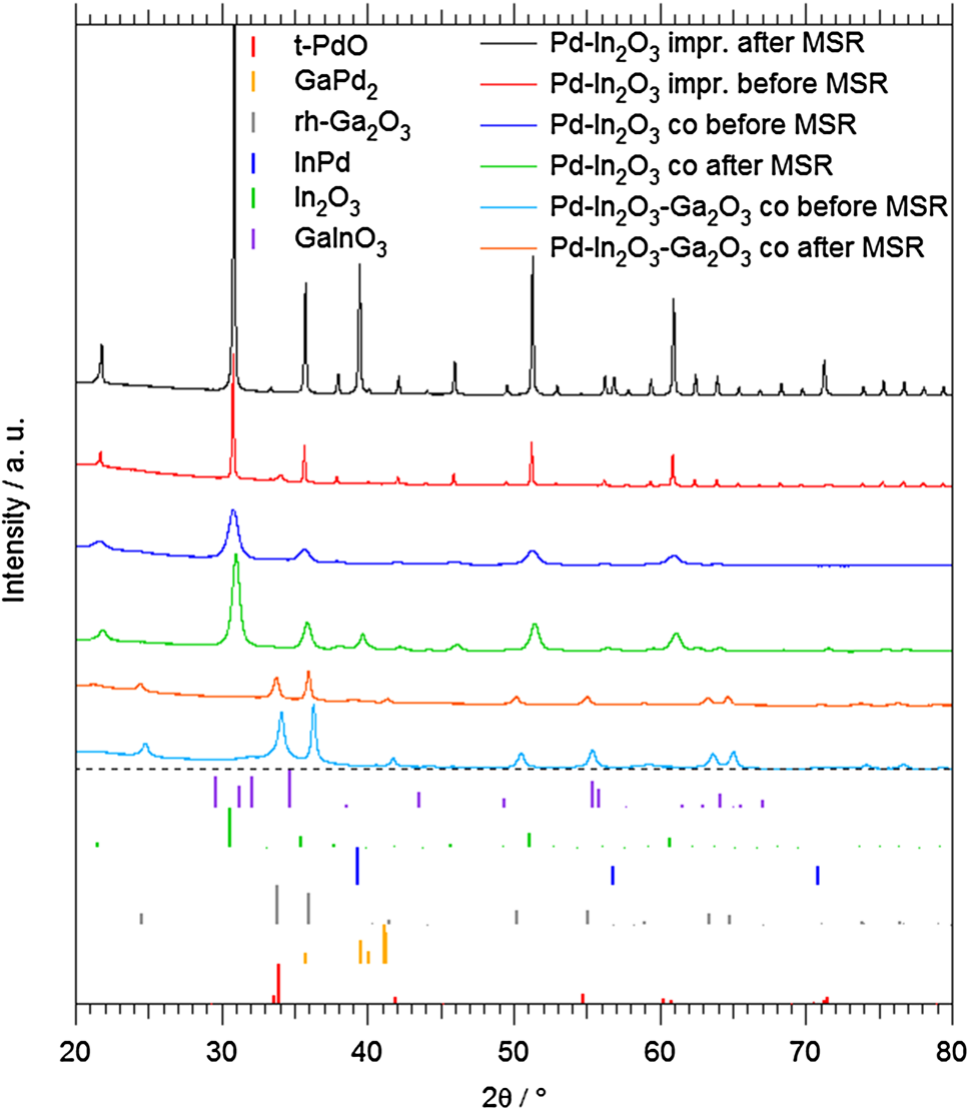
PXRD data of impregnated and co-precipitated Pd-In_2_O_3_ and co-precipitated Pd–Ga_2_O_3_–In_2_O_3_ before oxidation and after the catalytic MSR run. Reference diffractograms for tetragonal PdO (#43-1024), orthorhombic GaPd_2_ (#50-1443), rhombohedral Ga_2_O_3_ (#43-1013), cubic In_2_O_3_ (#06-0416), hexagonal GaInO_3_ (#21-0333) and cubic In_0.52_Pd_0.48_ (#46-1011) for phase analysis are shown as vertical bars. Data of the H_2_ pre-reduced state (after calcination and before MSR) are almost identical to those after MSR and are therefore not shown

All catalysts were subsequently characterized by X-ray diffraction (XRD) and (high resolution) electron microscopy (HRTEM) prior to and after methanol steam reforming. As the XRD (Figs. 1, 2, detailed discussion in Sect. 3.1.) and subsequent TEM analyses reveal, all three catalysts, after calcination, consist of Ga_2_O_3_ and In_2_O_3_ grains decorated with small PdO particles. To ensure similar starting conditions and to induce formation of the intermetallic compound/oxide interface, oxidative treatments (O_2_, 1 bar flowing, 673 K) and then activation in hydrogen stream (H_2_, 1 bar flowing, Pd–Ga_2_O_3_: 673 K; Pd–In_2_O_3_: 523 K; Pd–Ga_2_O_3_–In_2_O_3_: 473 K) were carried out prior to the actual catalytic measurement. Flow rates between 0.01 and 5.00 mL min^−1^ have been used.

### Catalytic Experiments

For all long-term catalytic measurements, a plug-flow reactor setup (PID Eng&Tech) was used. The flow reactor setup consists of a reactor core, which is represented by a 20 cm long steel cylinder, the inner walls of which are coated by silica to prevent influences by any spurious catalytic activity of steel. The catalyst is positioned within this cylinder using quartz glass wool. The steel tube is further located inside a furnace, allowing temperatures up to 773 K. At the upper gas inlet, a thermocouple is integrated, which extends down into the catalyst bed inside the reactor tube. The reactor itself is connected to the gas supply and discharge ports via Swagelok® quick connectors for easy removal and exchange of catalysts. The gas feed is provided by a constant flow in top-to-bottom direction over the catalyst bed. The gas inlet and outlet are connected to each other by a six-way valve, which serves as a bypass of the reactor section if needed. The various gases are introduced via mass-flow controllers, which makes a wide range of different gas mixtures available. Furthermore, a heated injection valve is located next to the inlet port, which is equipped with a Gilson HPLC pump, enabling liquids to be mixed with the gas stream after vaporization (flow rate 0.01–5.00 mL min^−1^). Most parts (except for gas analysis and external liquid pump) are placed inside a temperature-controlled area of 393 K to exclude condensation phenomena. For similar reasons, the injector as well as the gas-feed pre-heating unit are kept at elevated temperatures. After passing the reactor section, the gas stream exits the temperature-controlled area and, subsequently, all liquid contents (in this case methanol and water) are removed by a Peltier separating unit in addition to a Nafion© membrane only penetrable for gases. The dry gases are detected by a Varian micro-GC system consisting of three separate chromatography columns for hydrogen, carbon monoxide and carbon dioxide, respectively.

For all experiments, a methanol–water mixture of 1:1 composition was used under flowing conditions. The steam flow was set to 1 mL min^−1^ and mixed with the carrier-gas stream (8 mL min^−1^ N_2_/He mixture, the latter as internal standard) before entering the reactor section. The total pressure in the apparatus is limited to 1 bar. The reactor setup shows no conversion in MSR under the conditions applied.

As for the selectivity, no CH_4_ is observed. The CO-selectivity can be obtained by subtracting the selectivity to CO_2_ from 100%. Hydrogen selectivity is 100%, since no other hydrogen-containing product is detected.

### Structural Characterization

Powder X-ray diffraction was conducted on a STOE-STADIP-MP powder diffractometer in Bragg–Brentano geometry (Cu K_α1_-radiation, Ge(111) monochromator) from 2θ = 5° to 100°.

A Philips CM200FEG microscope operated at 200 kV and equipped with a field emission gun, Gatan imaging filter, and an energy-dispersive X-ray (EDX) analyser was used for TEM studies. The coefficient of spherical aberration was Cs = 1.35 mm, and the information limit was better than 0.18 nm. Selected areas were processed to obtain the power spectra (PS, square of the Fourier transform of the image), which were used for measuring interplanar distances (± 0.5%) and angles (± 0.5 deg) for phase identification. Projected areas have been measured and equivalent diameters calculated for a certain number of catalyst particles in each sample; in all cases (except for the impregnated Pd/Ga_2_O_3_) the values of standard error of the mean diameter were ≤ 0.3 nm. Frequency distributions of particle sizes fitted well to lognormal functions.

## Results and Discussion

### Structural Characterization

#### X-ray Diffraction

X-ray diffraction patterns collected for all catalysts before and after the catalytic treatments are highlighted in Figs. 1 and 2. For the impregnated/co-precipitated Pd–Ga_2_O_3_ catalysts (Fig. 1), similarities but also distinct differences arise. In the state before catalysis (i.e. before hydrogen pre-reduction and after oxidation in air), the impregnated Pd-Ga_2_O_3_ catalyst is composed of PdO and β-Ga_2_O_3_, which is expected since impregnation was performed on phase-pure commercial β-Ga_2_O_3_ powder (black diffractogram in Fig. 1 with majority of reflexes corresponding to lilac bars, PdO best visible at the characteristic split reflex at 2θ = 34°). After H_2_ pre-reduction and the subsequent long-term presence in the methanol steam reforming mixture, GaPd_2_ and mainly unaltered β-Ga_2_O_3_ are detected (red diffractogram). GaPd_2_ is also found on the co-precipitated catalyst after the pre-reduction/catalytic treatment, but the catalyst support structure before and after catalytic treatment is rhombohedral (α-)Ga_2_O_3_, which obviously results from the co-precipitation procedure favouring this polymorph. Remarkably, metastable rhombohedral Ga_2_O_3_ persists during each step of a catalytic cycle, that is, after pre-oxidation, pre-reduction and catalytic treatment (light grey bars/green and blue diffractograms in Fig. 1).

Structurally, the impregnated/co-precipitated Pd–In_2_O_3_ materials appear less complex (Fig. 2). In both cases, before oxidation, tetragonal PdO/cubic In_2_O_3_ is present. After the MSR treatment, the structure of the catalysts is characterized as InPd/cubic In_2_O_3_.

**Fig. 3:**
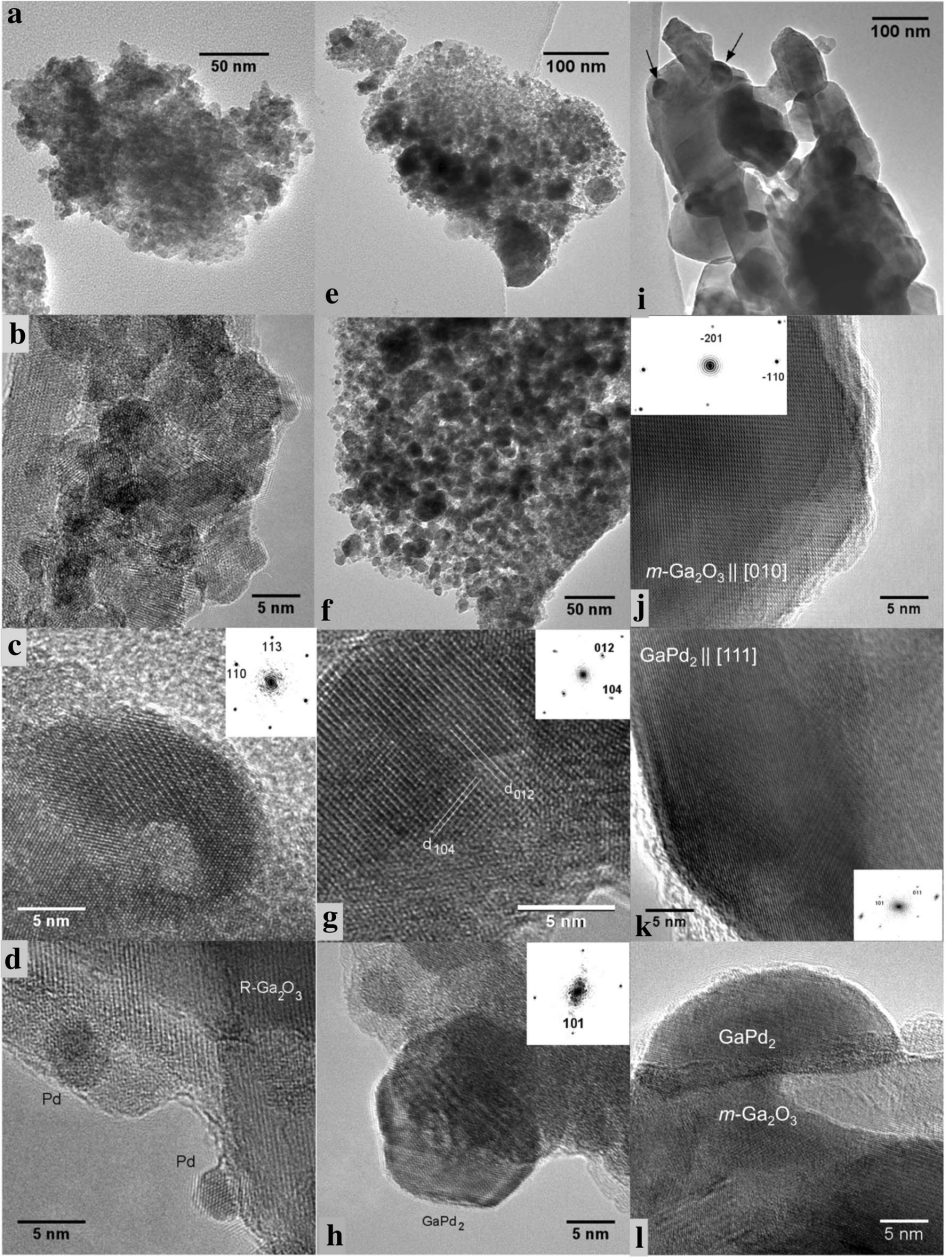
TEM and HRTEM images of the co-precipitated and impregnated Pd–Ga_2_O_3_ catalysts: **a***–***d** freshly prepared by co-precipitation and calcination in air at 773 K; **e***–***h** co-precipitated, after the MSR run; **i**–**l** impregnated, after the MSR run. Insets in **c, g, h, j, k** examples of power (FFT) spectra used for phase identification and determination of crystal orientation. Arrows in **i** show some of the GaPd_2_ particles

Apparently, the Pd–Ga_2_O_3_–In_2_O_3_ catalyst is the crystallographically most complex system (Fig. 2). Before oxidation, weak signals of PdO are present alongside those of cubic In_2_O_3_ and rhombohedral Ga_2_O_3_. After catalysis, the latter two are still present, in addition to very weak and broad signals of InPd. GaPd_2_ is not visible in the XRD patterns, but may partially overlap with the broad InPd reflection at 2θ = 39°. It is, however, locally detectable in HRTEM images (*cf*. Fig. 3f). The same is true for the hexagonal ternary oxide GaInO_3_, detected also by HRTEM, which appears to have formed during the co-precipitation process.

#### High-Resolution Electron Microscopy

EDX spectra (not shown) taken in 14 different areas reveal the mean ratio of Ga:Pd to be 80:20 ± 4 (std.err.) in the fresh PdO–Ga_2_O_3_ catalyst after calcination. Analyses of lattice spacings and angles in HRTEM images allow the unambiguous phase identification in most cases (examples are given in Fig. 3g, h, j, k). In general, the structure of the co-precipitated catalyst did not change dramatically during the catalytic reaction, except for the particle size (see Table 1) and their composition. Only some small Pd particles individually supported on rhombohedral gallium oxide (α-Ga_2_O_3_)—Fig. 3d—or those intermixed with differently sized oxide particles (Fig. 3b) have been observed in the fresh catalyst. Occasionally, amorphous material and gallium oxide particles with the cubic spinel-like structure have been detected. Monoclinic Ga_2_O_3_ as the thermodynamically most stable Ga_2_O_3_ polymorph appears to be absent at all stages. During reduction and subsequent reaction, elemental palladium is transformed to GaPd_2_ with much larger particle size (Fig. 3e, f, h). Frequently, the GaPd_2_ crystals display {101} and {001} facets (Fig. 3h). The surfaces of the GaPd_2_ particles are clean and not covered with any overlayers, but in several cases, single crystalline particles displayed inhomogeneous contrast of “core–shell” type (not shown) with darker cores and lighter shells—probably due to depletion of the sub-surface regions with Pd. This is supported by EDX analyses of individual intermetallic particles which show some deficiency of Pd with respect to the stoichiometric ratio Ga:Pd = 1:2 in most cases. Locally, Ga_7_Pd_3_ is also present as a minority phase in the catalyst after reaction. It is noteworthy that, in contrast to XRD, no Pd oxide was found with HRTEM in neither fresh, nor in the reacted material, probably because of its instability under the electron-beam in high vacuum.

**Table 1:** Overview of the structural and catalytic findings by HRTEM, XRD and methanol steam reforming

Support	Phases (XRD)	Phases (HRTEM)	Ga(In):Pd (EDX)	D_vol.wtd_ (Pd phase), nm (TEM)	Surface area (Pd phase) m^2^g^−1^ (TEM)	CO_2_ Selectivity (%)	Conversion (%)
Ga_2_O_3_	**rh-Ga**_**2**_**O**_**3**_/**t-PdO**	**rh** + **c-Ga**_**2**_**O**_**3**_/**Pd**		**3.2**	**25.6**		
	rh-Ga_2_O_3_/GaPd_2_	rh + c-Ga_2_O_3_/GaPd_2_	47:53	18.6	4.4	90	82
	*m-Ga*_*2*_*O*_*3*_/*GaPd*_*2*_	*m-Ga*_*2*_*O*_*3*_/*GaPd*_*2*_	*39:61*	*88.4*	*0.9*	*59*	*58*
In_2_O_3_	**c-In**_**2**_**O**_**3**_/**t-PdO**	**c-In**_**2**_**O**_**3**_/**Pd**		**5.6**	**14.7**		
	c-In_2_O_3_/InPd	c-In_2_O_3_/InPd	51:49	23.5	3.5	97.5	91
	*c-In*_*2*_*O*_*3*_/*t-PdO*	*c-In*_*2*_*O*_*3*_/*InPd, InPd*_*2*_	*46:54*	*12.7*	*6.5*	*99*	*50*
Ga_2_O_3_/In_2_O_3_	**rh-Ga**_**2**_**O**_**3**_/**t-PdO**	**rh-Ga**_**2**_**O**_**3**_/**rh-In**_**2**_**O**_**3**_/**h-GaInO**_**3**_/**Pd**/**GaPd**_**2**_	n/a	**4.1**	**20.1**		
	rh-Ga_2_O_3_/t-PdO/InPd	rh-Ga_2_O_3_/c-In_2_O_3_/GaPd_2_/InPd		8.5	9.6	97.5	60

Bold values represents fresh co-precipitated catalyst

Normal values represents co-precipitated catalyst after MSR reaction

Italic values represents impregnated catalyst after MSR reaction

In contrast to the co-precipitated materials, where only rhombohedral Ga_2_O_3_ was found, in the impregnated catalysts, large single-crystalline particles (sometimes of submicrometer size) of the monoclinic Ga_2_O_3_ polymorph (with elemental Pd particles decorating them prior to reduction/catalysis) were covered with GaPd_2_ after reduction/catalysis—Fig. 3i–k. The particles exhibit a tendency to wet the surface of the support, displaying some kind of metal-support interaction—Fig. 3l. On average, the GaPd_2_ particles are closer to the stoichiometric composition, according to EDX, but nevertheless did not correspond exactly to the nominal formula.

**Fig. 4:**
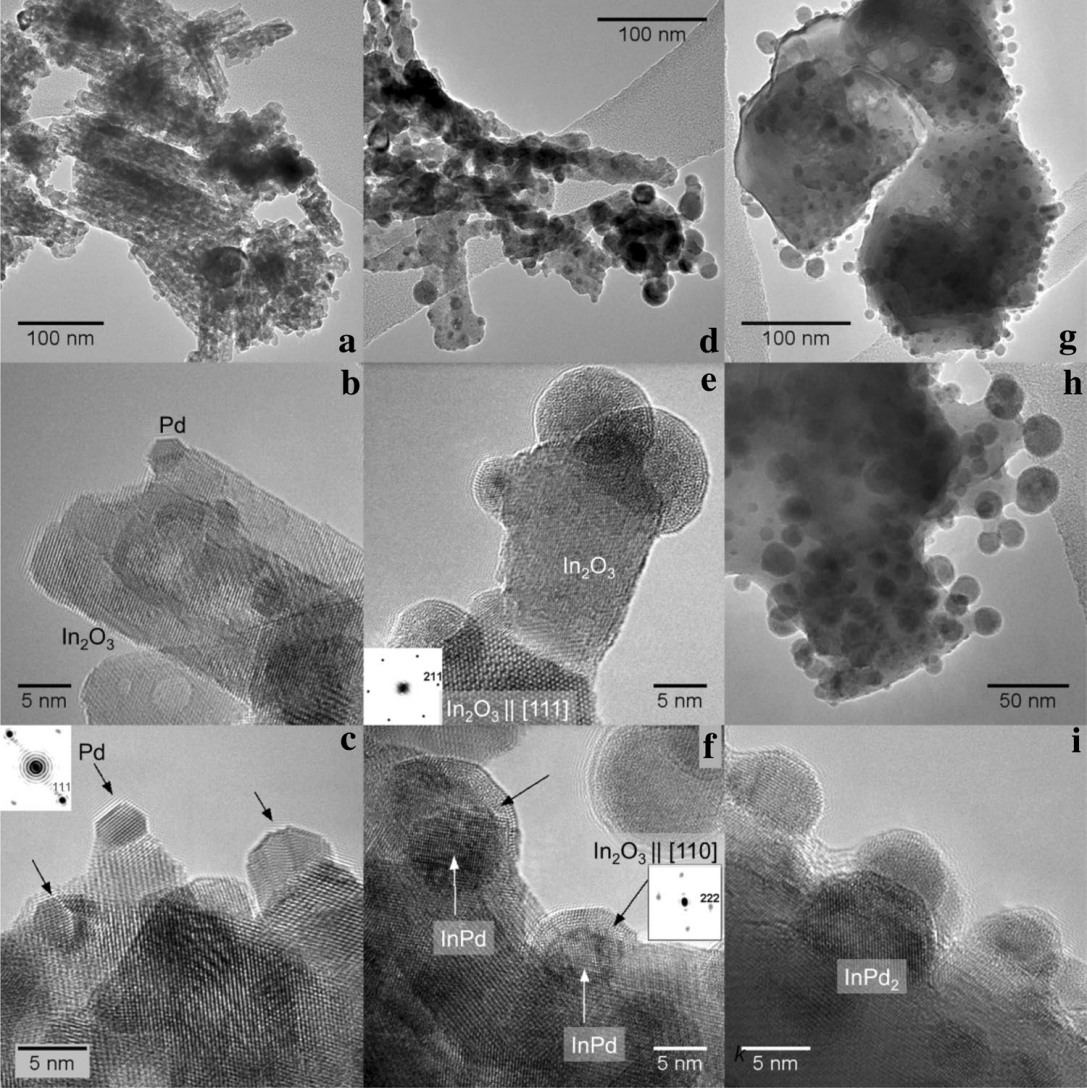
TEM and HRTEM images of co-precipitated and impregnated Pd–In_2_O_3_ catalysts: **a**–**c** freshly prepared by co-precipitation and calcination in air at 773 K; **d***–***f** co-precipitated, after the MSR run; **g**–**i** impregnated, after the MSR run. Insets in **c, e, f** examples of power (FFT) spectra used for phase identification and determination of crystal orientation of indicated phases. Black arrows in **c** point to Pd particles, in **f** to In_2_O_3_ shells on InPd particles. Elemental Pd in the fresh catalyst is due to e-beam reduction of PdO in the electron microscope

EDX spectra (not shown) taken in eight different areas yield a mean ratio of In:Pd = 83:17 ± 5 (std.err.) in the fresh co-precipitated and calcined Pd-In_2_O_3_ catalyst. Both phases (elemental Pd and bcc In_2_O_3_) are well crystallized (Fig. 4a–c); no amorphous material was found. Pd particles are distributed on the In_2_O_3_ surfaces without clustering. They display clean surfaces, sharp edges and, quite frequently, twin boundaries (Fig. 4b, c)—a feature that is known to increase the activity of Cu in the industrial Cu/ZnO/Al_2_O_3_ catalyst [25]. In addition to pure Pd, a few particles of InPd_2_ have been locally detected in the spent impregnated catalyst, highlighting the increased intermixing ability of Pd and In.

In most cases, the identification of Pd-containing phases from HRTEM data is ambiguous for the Pd–In_2_O_3_ catalyst studied after the MSR run, because of the strongly distorted structure of the particles—especially in the impregnated system—that could probably be caused by non-stoichiometry (variable In/Pd ratio). Nevertheless, the structure of InPd fits the majority of the HRTEM images in the co-precipitated system, and InPd_2_ for the impregnated catalyst. This is in agreement with XRD (except for InPd_2_). On average, EDX analyses of individual particles reveal the compositions to be close to the stoichiometric composition of InPd in the co-precipitated catalyst after the reaction, but the In:Pd ratio was shifted towards Pd in the impregnated material. Both types of particles partially exhibit shells of distorted In_2_O_3_ (Fig. 4e, f, i), which are thicker in the co-precipitated (up to 2 nm), and thinner and less-ordered in the impregnated catalyst.

**Fig. 5:**
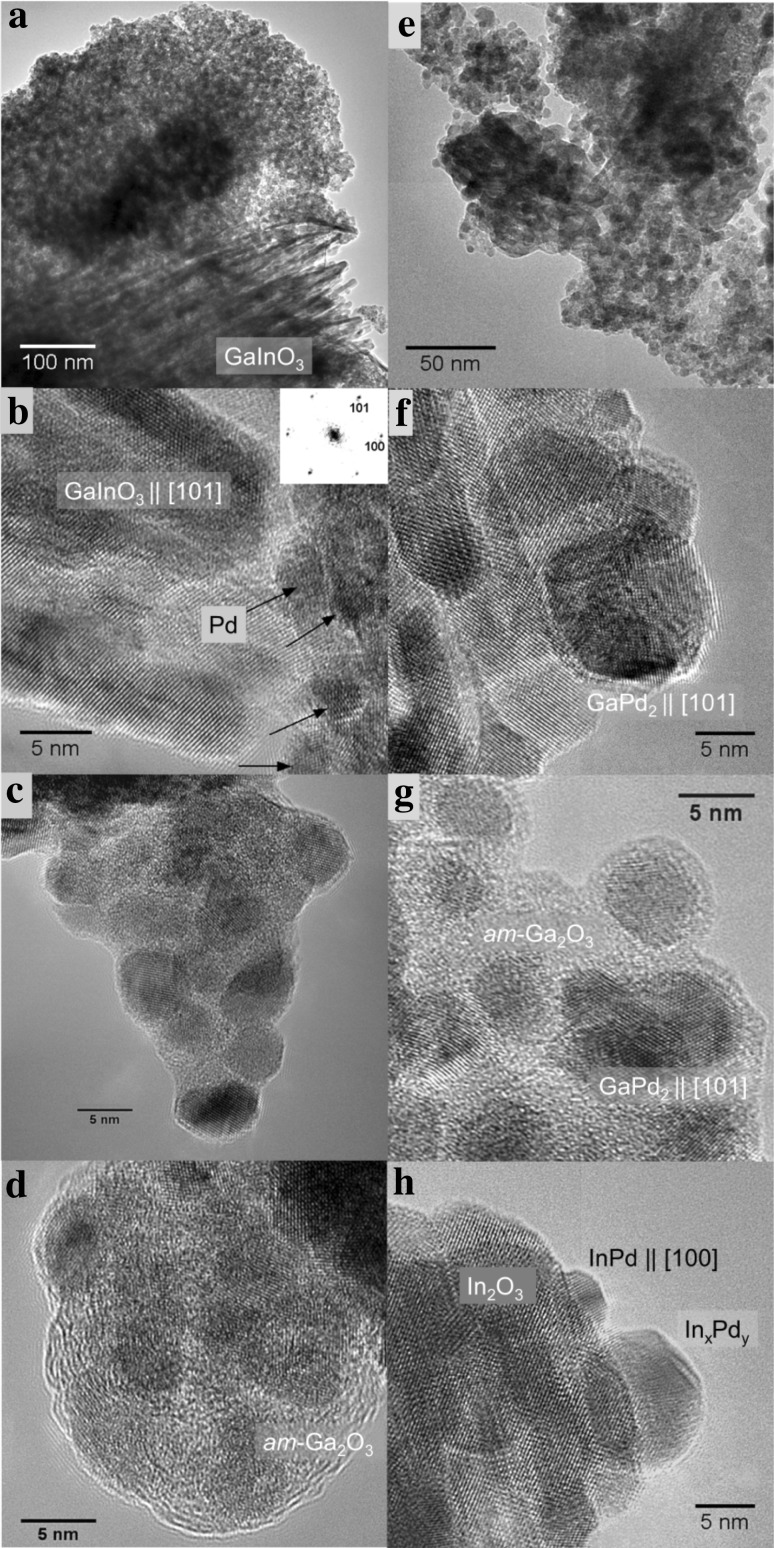
TEM and HRTEM images of the co-precipitated Pd–Ga_2_O_3_-In_2_O_3_ catalyst **a**–**d** freshly prepared by co-precipitation and calcination in air at 773 K; **e***–***h** co-precipitated, after the MSR run. Inset in **b** example of powder spectra used for phase identification and determination of crystal orientation. Black arrows in **b** point to Pd particles. Elemental Pd in the fresh catalyst is due to e-beam reduction of PdO in the electron microscope

For the Pd–Ga_2_O_3_–In_2_O_3_ system, the composition could be determined by EDX to be Ga:In:Pd = 65:17:18 ± 2 (std.err.) in the fresh Pd–Ga_2_O_3_–In_2_O_3_ material. Along with the individual gallium and indium oxides, the hexagonal phase of GaInO_3_ has been detected with HRTEM in freshly prepared mixed-oxide supported catalyst (Fig. 5a, b). The palladium particles in the fresh catalyst were similar to those in the single-oxide materials: they exhibited a cuboctahedral shape.

After reaction, the majority of Pd-containing particles have been identified as GaPd_2_ and InPd (or In_x_Pd_y_ with probably variable In:Pd ratio)—Fig. 5 b, f–h. The former were typically embedded in an amorphous matrix (similar to Pd in the freshly prepared catalyst)—Fig. 5c, d, g—and displayed shells (also amorphous), but the latter had clean surfaces (Fig. 5h). In the fresh catalyst, several particles of the rhombohedral polymorph of In_2_O_3_ have been found. Table 1 quickly summarizes the main findings of the XRD and TEM work in direct comparison with the catalytic findings, discussed in Sect. 3.2.

### Catalytic Characterization in Methanol Steam Reforming

**Fig. 6:**
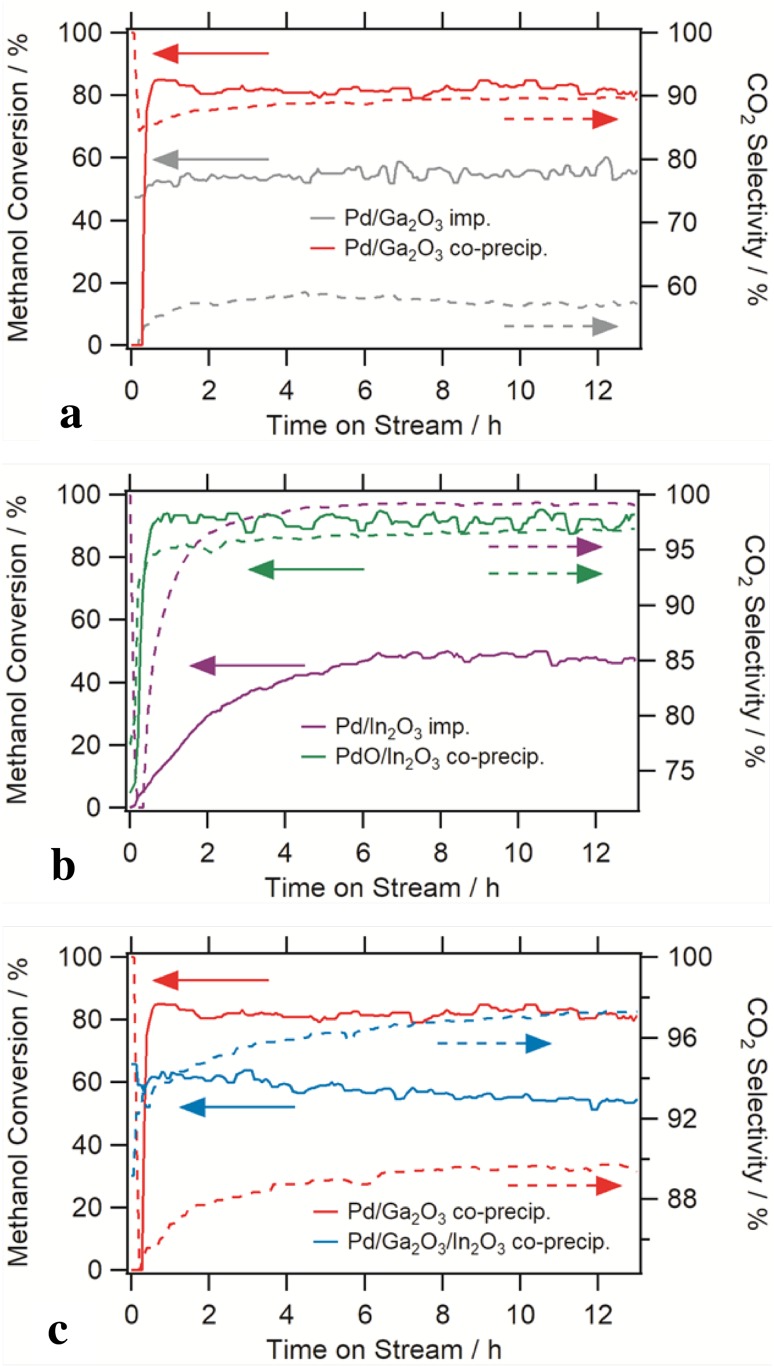
Comparative methanol steam reforming reaction profiles measured on a set of Pd–Ga_2_O_3_, Pd–In_2_O_3_ and Pd–Ga_2_O_3_–In_2_O_3_ catalysts. **a** shows the comparison of methanol conversion and CO_2_ selectivity vs. time-on-stream between a conventionally impregnated Pd-Ga_2_O_3_ catalysts and a co-precipitated one. In **b** the same is highlighted for the Pd-In_2_O_3_ systems. **c** Finally shows the comparison between an In_2_O_3_-doped Pd–Ga_2_O_3_ catalyst and an accordingly In_2_O_3_-free material. Prior to the methanol steam reforming reaction (molar ratio methanol:water = 1:1) at 573 K, pre-oxidation at 673 K in oxygen for 1 h, as well as pre-reduction in hydrogen (1 h) has been performed. Pre-reduction for Pd–In_2_O_3_ and Pd–Ga_2_O_3_ 523 K, for Pd–Ga_2_O_3_–In_2_O_3_ 473 K. Solid lines represent conversion, dashed lines refer to CO_2_-selectivity

The respective catalytic patterns are shown in Fig. 6. For all of the studied catalytic materials, the methanol conversion and CO_2_ selectivity are shown as a function of the time-on-stream. To focus on eventual deactivation, long-term experiments up to 12 h time-on-stream have been performed. In order to highlight possible activity and selectivity improvements by the co-precipitation preparation, the catalytic properties are directly compared to those of already well established impregnated materials.

The catalytic data in Fig. 6a for the Pd–Ga_2_O_3_ materials show that in contrast to the impregnated Pd–Ga_2_O_3_ catalyst, which exhibits a comparably low activity (58% methanol conversion) and a low CO_2_ selectivity (~ 59%), the co-precipitated Pd-Ga_2_O_3_ catalyst displays a much higher CO_2_ selectivity at around 90% (increasing from 88 to 91% in the course of the reaction), with an at the same time also much improved conversion (82%). Deactivation on the time scale of the experiment is clearly absent for both systems. The observed catalytic patterns are interesting from a structural point of view, since both the HRTEM and the XRD data reveal that in both cases, a GaPd_2_ phase has formed after reduction/during the catalytic treatment. Slight compositional variations have been monitored, but without structural breakdown of the GaPd_2_ phases. The results therefore directly prove what has already been derived from corresponding studies on support-free bulk GaPd_2_ intermetallic compounds:[8] the mere presence of the GaPd_2_ intermetallic compound is not enough to enable a high CO_2_ selectivity in methanol steam reforming. Rather, a synergistic action between intermetallic and oxidic support material must take place to enhance the water splitting capability of the intermetallic-oxide interface. Exactly this beneficial interface seems to be formed by co-precipitation, which then directly gives rise to improved CO_2_ selectivity. In the Pd–Ga_2_O_3_ case, co-precipitation also causes the exclusive formation of a GaPd_2_-rhombohedral Ga_2_O_3_ (α-Ga_2_O_3_) interface, which apparently is also active and selective in methanol steam reforming. The presented catalytic properties of this co-precipitated GaPd_2_/α-Ga_2_O_3_ material moreover strongly resemble similar studies of GaPd_2_/α-Ga_2_O_3_ catalysts, where α-Ga_2_O_3_ was impregnated by small Pd particles and subsequently subjected to hydrogen reduction to induce the formation of GaPd_2_ intermetallic particles [8]. The increase of activity and selectivity could also be explained by remnants of elemental Pd after pre-reduction, which are successively transformed into GaPd_2_ during methanol steam reforming (which might be easier if the metal-oxide interface is more extended).

A slightly different pattern is observed for the Pd–In_2_O_3_ catalysts (Fig. 6b). Here, the structural situation of both co-precipitated and impregnated catalysts is less complex, since, in both cases, after reduction and during catalysis, only cubic In_2_O_3_ and (compositionally slightly variable) InPd as the main phases are present. This is then directly reflected in the trends of the activity and CO_2_ selectivity. In both cases, CO_2_ selectivities of > 95% have been obtained. The methanol conversion of the co-preciptated InPd/In_2_O_3_ catalyst, is however, much higher (90 vs. 40% on the impregnated one).

Finally, Fig. 6c reveals how the CO_2_ selectivity of a co-precipitated GaPd_2_/Ga_2_O_3_ can be directly improved by promotion with In_2_O_3_. In comparison to the former, the CO_2_ selectivity of the latter can be improved from 90 to almost 98%. The methanol conversion of the In_2_O_3_-doped material at 60% is somewhat lower than that of the undoped sample (80%) and decreases slightly in the course of the reaction, indicating some deactivation.

## Conclusions

We have shown how the catalytic properties of already well-established intermetallic methanol steam reforming catalysts on Pd basis, namely GaPd_2_/Ga_2_O_3_ and InPd/In_2_O_3_, can be steered and exemplarily improved by a co-precipitation approach to synergistically alter the intermetallic compound-supporting oxide interface. This is especially evident for the GaPd_2_–Ga_2_O_3_ catalyst, where the use of co-precipitation gives rise to a selectivity improvement also for the undoped GaPd_2_ sample, which can be further beneficially influenced by In_2_O_3_ doping. This improvement can be achieved despite the increased structural complexity and chaotic morphology of the GaPd_2_/InPd/Ga_2_O_3_/In_2_O_3_ material. Apparently, the presence of a variety of distinct support- and intermetallic particle phases is not detrimental to activity/selectivity as long as the appropriate intermetallic phases are present and exhibit optimized intermetallic-support phase boundary dimensions.
